# Feasibility and Acceptability of an Incentive‐Based Intervention for Health Behaviour Change Among Rural Residents in China: A Mixed‐Methods Evaluation

**DOI:** 10.1111/hex.70518

**Published:** 2025-12-11

**Authors:** Bo Yan, Zhenke He, Ran Zhang, Bin Shao, Shuai Zheng, Xiaomin Wang, Xudong Zhou

**Affiliations:** ^1^ Institute of Social Medicine, School of Medicine Zhejiang University Hangzhou China; ^2^ School of Medicine Emory University Atlanta USA; ^3^ Changxing County Center for Disease Prevention and Control Huzhou China; ^4^ School of Public Administration Hangzhou Normal University Hangzhou China; ^5^ The Second Affiliated Hospital Zhejiang University School of Medicine Hangzhou China

**Keywords:** acceptability, digital platform, feasibility, financial incentives, healthy behaviours

## Abstract

**Background:**

Encouraging healthy behaviours among rural residents in China is crucial to promote health and well‐being. Although financial incentives are known to be effective in promoting single healthy behaviours, their efficacy in simultaneously encouraging multiple healthy behaviours remains unclear. This study aimed to test the feasibility and acceptability of an incentive‐based intervention to encourage multiple healthy behaviours.

**Methods:**

‘Health Bank’, a digital platform established by the Changxing County Center for Disease Control and Prevention, was employed financial incentives to engage villagers in promoting healthy behaviours. The intervention, conducted from January to October 2022, was followed by a mixed‐methods evaluation in November 2022 to assess its feasibility and acceptability as the primary outcomes. Feasibility was assessed based on quantitative data from self‐administered questionnaires and routine data, while acceptability was assessed by conducting semi‐structured interviews.

**Results:**

Routine data indicated that 1164 out of 3137 (37.1%) villagers participated in ‘Health Bank’, with the majority completing the required three daily healthy behaviours, despite their low participation rates in other behaviours. Among the 140 surveyed villagers, 68 respondents were not willing to participate in ‘Health Bank’, with only 19% refraining due to a lack of interest or need. The qualitative results suggested that financial incentives were a compelling factor for most participants, though challenges remained regarding the programme's elderly‐friendliness, gift redemption process and the range of activities covered.

**Conclusions:**

Although the incentive‐based behaviour change intervention exhibited promising feasibility and acceptability, participant feedback necessitates further modifications. To comprehensively assess the efficacy of this intervention, larger‐scale, randomised controlled trials were encouraged.

**Patient or Public Contribution:**

A team‐based approach was taken for developing the incentive‐based intervention model for this study. This included experienced staff from local Centers for Disease Control and Prevention, as well as community workers and community residents.

**Abbreviations**
BMI: body mass index
CCT: conditional cash transfer
CDC: Center for Disease Control and Prevention
GC: group contingency
PB‐CM: probability‐based cost management.

## 1 Introduction

Unhealthy behaviours have been identified as major contributors to chronic diseases and premature mortality [1, 2, 3, 4], increasing the risk of common diseases, such as obesity, diabetes and cancer, inflicting substantial economic burdens and immeasurable suffering [5]. In China, studies have shown that more than 50% of disease burdens associated with chronic diseases can be effectively mitigated by changes in healthy behaviours [6]. However, despite efforts to promote healthy behaviours, unhealthy behaviours remain prevalent [7]. According to the 2022 National Health Literacy Survey, only 27.8% of the population met the criteria for basic health literacy. Among rural residents, this proportion was substantially lower, at 23.8% [8].

Previous studies have identified economic and income issues as the primary drivers of health inequalities in rural China [9]. Relative income deprivation among rural residents has been shown to significantly exacerbate health disparities [10]. Lower economic status is associated with a higher probability of falling into poverty due to medical expenditures, which in turn leads to adverse health behaviours such as reduced investment in preventive health measures and delays in seeking medical care [11]. These findings suggest that financial incentives could serve as a potentially effective strategy to promote healthier behaviours among rural populations.

Financial incentive interventions have been widely used to promote health behaviours and improve health outcomes. Empirical studies have demonstrated its effectiveness in encouraging physical activity among young people, supporting weight loss in individuals with obesity, reducing smoking, preventing and managing chronic diseases, and increasing vaccination uptake [12, 13, 14, 15, 16, 17, 18]. Among these approaches, conditional cash transfers (CCTs) have gained increasing popularity as a means of enhancing engagement with health services. CCT programmes provide cash payments to low‐income households contingent upon predefined actions, thereby addressing demand‐side barriers [19]. CCTs have demonstrated success in promoting behaviours such as uptake of emerging vaccines, regular visits to health facilities, consistent school attendance among children, and adherence to health and nutrition promotion activities [19, 20, 21]. However, instances of ineffectiveness have also been documented [22, 23]. Thus, we are interested in examining the effectiveness of CCTs in rural China.

In impoverished rural areas of central and western China, CCTs have been effectively employed to improve the uptake of maternal and child health services, reduce school dropout rates among junior high school students from poor households, and enhance caregivers' nutritional knowledge and feeding practices [24, 25, 26]. However, existing studies have typically targeted single specific behaviours, failing to assess the impact of different incentive combinations on multiple health behaviours within a single study. Previous research has called for more comparative investigations into various components of financial incentives, including incentive magnitude, delivery mechanisms and the use of non‐monetary incentives [27]. Bachireddy et al. found that varying financial incentive structures could effectively promote sustainable changes in health behaviours; however, they also noted that over‐complicated incentive designs might exceed participants' information processing capacity or diminish their motivation to engage with the intervention [28]. Therefore, our study also aimed to explore the rationale underlying existing incentive approaches to inform the design of more acceptable intervention strategies in the future.

Moreover, regarding the health behaviours included, about half of adults and a fifth of children are overweight or obese according to the Chinese criteria [29]. Among adults over 18 years of age, the prevalence of hypertension and diabetes reached 23.2% [30] and 12.8% [31], respectively. Of this population, only 12.5% reported regular leisure‐time physical activity [32], and 27.78% have qualified overall health literacy [8]. In the light of these findings, this study included these significant health behaviours to incorporate, including physical activity, vaccination, weight control, chronic disease monitoring, attendance at health lectures and testing of health‐related knowledge.

In summary, the primary objective of this study is to evaluate the feasibility and acceptability of an incentive‐based intervention for promoting healthy behaviours. Furthermore, digital technologies have been successfully applied to behavioural domains, showing positive societal impacts and promise in the effectiveness and scalability of intervention [33, 34]. Therefore, the secondary objective is to assess the acceptability of a digital platform utilised to expand the scope of the intervention and reduce labour costs.

## 2 Methods

### 2.1 Study Design and Setting

The intervention was conducted in Xiangyang Village, Changxing County, Zhejiang Province, spanning 10 months between January and October 2022. The village has a population of approximately 3200 [35], and the prevalence of hypertension and diabetes is about 22.1% and 6.1%, respectively [36]. There are two rural doctors in the village who provide services such as primary care and chronic disease management, including regular measurement of blood pressure and blood glucose, and health education. To evaluate the feasibility and acceptability of the intervention, we applied mixed methods in November 2022, conducting quantitative and qualitative research. The quantitative survey utilised a self‐administered questionnaire (Supporting Information 2, Supporting Information 3), and the qualitative study included semi‐structured interviews (Supporting Information 4) with various stakeholders. In addition, routine data for the first 10 months of intervention were collected from the local Center for Disease Control and Prevention (CDC). No external health campaigns were implemented at the pilot site during the same period.

### 2.2 Trial Description (Digital Platform‐Based Interventions)

**Figure 1:**
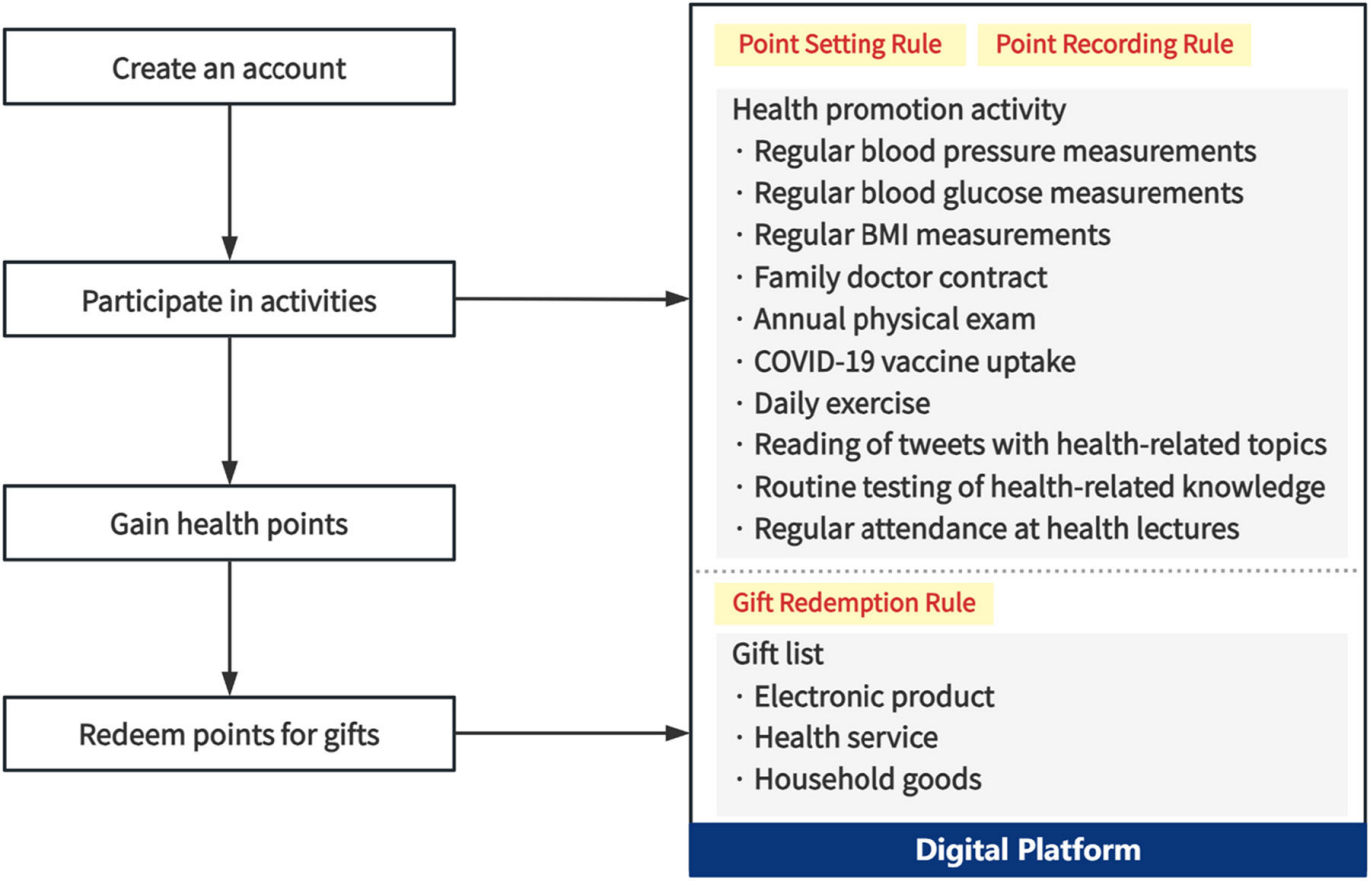
Implementation mode of ‘Health Bank’.

‘Health Bank’ is a digital platform established by the Changxing County CDC to engage villagers in promoting healthy behaviours through financial incentives. Everyone was eligible to participate in the ‘Health Bank’, and those without mobile phones could be involved with the assistance of family members or volunteers. Publicity campaigns were conducted primarily through official social media channels of CDC, television programmes, short‐form video platforms and community‐based outreach efforts by local health workers. Figure 1 presents an overview of selected healthy behaviours, including: (1) regular blood pressure, blood sugar, and body mass index (BMI) measurements; (2) annual physical exam; (3) Covid‐19 vaccine uptake; (4) daily exercise; (5) reading of tweets with health‐related topics; (6) routine testing of health‐related knowledge; and (7) regular attendance at health lectures. In addition, the intervention incorporated the ‘Family Doctor Contract Services’, a government health service accessibility programme that aims to establish a long‐term and stable relationship between general practitioners (in this study, village doctors) and families by signing a contract to protect their health throughout the entire process [37].

Each behaviour was assigned a corresponding score for completion, and specific scoring principles are presented in Table S1 (Supporting Information 1). Healthy behaviour score records consisted of two categories: (1) automatic entry online and (2) manual entry offline. With informed consent, villagers created a personal account on the digital platform. Participants engaging in automatically online‐recorded behaviours such as daily exercise, reading of tweets with health‐related topics, and routine testing of health‐related knowledge had their participation data automatically recorded by the ‘Health Bank’ platform, which were then converted into corresponding incentive points. For instance, participants who achieved a daily step count of at least 6000 steps on a minimum of 18 days per month had their step counts automatically recorded by the ‘Health Bank’ platform. These individuals were allocated 5 points monthly for fulfilling this criterion. Similarly, completion of one health‐related tweet reading within the platform was rewarded with 1 point, while each completed health knowledge assessment yielded 5 points. For activities requiring manual entry, including regular blood pressure, regular attendance at health lectures or Covid‐19 vaccine uptake, points were verified and manually entered into participant accounts by village doctors. Active participants were defined as those who achieved a total score exceeding 50 points between January and October 2022, having attained the 50‐point threshold through completion of three strongly recommended foundational activities: Covid‐19 vaccine uptake, family doctor contract and annual physical exam.

Table S2 (Supporting Information 1) showed the gift redemption rules of ‘Health Bank’ in detail. In the gift redemption pool, participants can redeem their scores for a variety of gifts with distinct values, including household goods, electronic products and health services. The more scores, the higher value of the gift. Used scores were cleared after each redemption.

### 2.3 Data Collection

#### 2.3.1 Quantitative Data

The survey respondents were all permanent villagers of Xiangyang Village. Both participants and non‐participants of ‘Health Bank’ were included in the quantitative survey. Those with comprehension difficulties responding to questions, such as illiteracy, were excluded. According to the latest 2020 census data, 91.6% of the county population has a primary school education level or above, with an illiteracy rate of merely 2.19% [38]. A total of 140 eligible participants were recruited using convenience sampling, with 34 participating ‘Health Bank’. Convenience sampling was deemed appropriate during the initial pilot phase to ensure timely and cost‐effective assessment of the intervention's feasibility. Recruitment was carried out by village staff and village doctors disseminating information through relevant social media groups. All of them were informed that their participation was confidential, voluntary and could be withdrawn at any time.

#### 2.3.2 Routine Data

Information on 1164 villagers who participated in ‘Health Bank’ between January and October 2022 was obtained from the basic information database provided by the Changxing County CDC, including socio‐demographic characteristics, the incidence of diabetes and hypertension, as well as the frequency and score of villagers' participation in each selected healthy behaviour in the ‘Health Bank’.

#### 2.3.3 Qualitative Data

A purposeful sampling strategy was employed to recruit villagers to gain insights from different perspectives. The selection criteria were not confined to age or gender, although efforts were made to maintain a balanced demographic distribution. The semi‐structured interviews were conducted with 16 respondents, of whom nine were over 50 years of age, eight were male, five were villagers who participated in the ‘Health Bank’, five were villagers who did not participate in the ‘Health Bank’, two were village doctors, two were village heads, and two were CDC staffs. Interviews were conducted by the principal investigator at the local community health centre. Each face‐to‐face interview lasted approximately 15–20 min, was audio‐recorded in its entirety, and subsequently underwent verbatim transcription. All participants provided verbal informed consent before the interviews.

### 2.4 Ethical Considerations

This evaluation study, conducted in collaboration with the local government, focused on the ‘Health Bank’ platform developed and managed by the local CDC, with written informed consent obtained from all participants. In China, data from health sectors are accessible to research and utilised for participant identification and contact. Therefore, a formal exemption from ethical approval was granted for this study, as the evaluation did not limit the participants' autonomy in deciding to participate in the ‘Health Bank’. Due to the retrospective nature of the study, the Ethics Committee waived the requirement of ethical approval. We followed ethical principles by guaranteeing the confidentiality and privacy of participants' information. The study was conducted in accordance with the relevant guidelines and regulations for non‐clinical trials.

### 2.5 Measures

Quantitative research was conducted with our self‐administered questionnaire, which consisted of three domains: (1) socio‐demographic characteristics, including gender, age, average monthly household income, education level and health status; (2) information channels and participation status of ‘Health Bank’; and (3) willingness and suggestions for the promotion of ‘Health Bank’.

Different sets of questions were designed for various qualitative interviewees. For villagers, we investigated their awareness of ‘Health Bank’ and barriers to participation. For village doctors, village heads, and CDC staffs, we focused on their attitudes towards difficulties in implementing ‘Health Bank’ and their opinions on its further development from a provider's perspective. All interviews lasted between 10 and 30 min and were audio‐recorded and transcribed verbatim.

### 2.6 Outcomes

The study focused on two primary outcomes: feasibility and acceptability. Feasibility was investigated quantitatively, and acceptability was investigated using both quantitative and qualitative methods.

Feasibility was assessed by: (1) the number of active ‘Health Bank’ participants; (2) socio‐demographic characteristics of 140 survey participants and 1164 participants from routine data; and (3) attitudes towards difficulties in implementing ‘Health Bank’.

Acceptability was assessed by: (1) reasons for not participating in ‘Health Bank’; (2) awareness of ‘Health Bank’; (3) attitudes towards the shortcomings of ‘Health Bank’; (4) suggestions for the improvement of ‘Health Bank’ and (5) attitudes towards financial incentives. Additionally, we examined participants' attitudes towards the digital platform as a secondary aim to assess the acceptability of our digital platform.

### 2.7 Statistical Analysis

For quantitative data, descriptive analyses were performed to summarise the weighted frequencies and percentages of individual characteristics. *χ* ^2^ tests were conducted to examine the differences in socio‐demographic characteristics between villagers who participated in ‘Health Bank’ and those who did not, based on survey data, as well as between active participants and inactive participants based on routine data. All statistical analyses were performed using SPSS version 26.0 and R version 4.2.1, with a statistical significance level of *p* < 0.05.

For qualitative interview data, transcripts were reviewed and independently coded by two researchers. All disagreements were agreed upon through discussion among all authors. We generated a list of themes using an inductive thematic analysis [39] to identify limitations and improvements related to ‘Health Bank’. We used Microsoft Word and Excel version 21.0 (Microsoft Corp) for managing the data.

## 3 Results

### 3.1 Feasibility

#### 3.1.1 Survey Data (140 Survey Respondents)

##### 3.1.1.1 Quantitative Findings of Survey Data

Table 1 shows that among the 140 survey respondents, 24.3% participated in ‘Health Bank’. Most of the ‘Health Bank’ participants surveyed were female, aged 45–59, with an average monthly household income of less than 6000 RMB (857 US dollars), with an education level of primary school or below, and were involved in family doctor contract services. Compared with men, women were two times more likely to participate in ‘Health Bank’ (32.9% vs. 13.1%, *p* = 0.007). The 45–59 age group demonstrated higher involvement in ‘Health Bank’ (*p* = 0.011). Those who contracted a family doctor were five times more likely to participate in ‘Health Bank’ compared to those who did not contract a family doctor (33.0% vs. 6.5%, *p* < 0.001). Those who received information from family doctors or village heads were significantly more likely to participate (*p* < 0.001). In addition, those who had access to a smartphone were more likely to participate in ‘Health Bank’ than those who did not (32.6% vs. 8.3%, *p* < 0.001).

**Table 1:** Characteristics of the survey respondents stratified by participation or not in ‘Health Bank’ (*N* = 140).

Characteristics	Sample population, *N* (%)^a^				
	Total	Participant	Non‐participant	*χ* ^2^	*p* b
Gender				7.336	0.007
Male	61 (43.6)	8 (23.5)	53 (50.0)		
Female	79 (56.4)	26 (76.5)	53 (50.0)		
Age group				11.184	0.011
< 45	15 (10.7)	3 (8.8)	12 (11.3)		
45–59	53 (37.9)	21 (61.8)	32 (30.2)		
60–74	56 (40.0)	8 (23.5)	48 (45.3)		
≥ 75	16 (11.4)	2 (5.9)	14 (13.2)		
Average monthly household income (RMB)				9.392	0.094
≤ 2000	49 (35.0)	6 (17.6)	43 (40.6)		
2001–4000	19 (13.6)	8 (23.5)	11 (10.4)		
4001–6000	21 (15.0)	6 (17.6)	15 (14.2)		
6001–8000	12 (8.6)	5 (14.7)	7 (6.6)		
8001–10,000	12 (8.6)	3 (8.8)	9 (8.5)		
> 10,000	27 (19.3)	6 (17.6)	21 (19.8)		
Education level				2.399	0.494
None	27 (19.3)	4 (11.8)	23 (21.7)		
Primary school	62 (44.3)	17 (50.0)	45 (42.5)		
Middle school	36 (25.7)	8 (23.5)	28 (26.4)		
High school and above	15 (10.7)	5 (14.7)	10 (9.4)		
Information channel				67.347	< 0.001
Village doctors	36 (25.7)	23 (67.6)	13 (12.3)		
Village heads	11 (7.9)	5 (14.7)	6 (5.7)		
Relatives, neighbours or friends	8 (5.7)	1 (2.9)	7 (6.6)		
CDC staff	10 (7.1)	5 (14.7)	5 (4.7)		
Billboards	6 (4.3)	0 (0)	6 (5.7)		
Not heard of	69 (49.3)	0 (0)	69 (65.1)		
Hypertension or diabetes				0.001	0.973
Absent	82 (58.6)	20 (58.8)	62 (58.5)		
Present	58 (41.4)	14 (41.2)	44 (41.5)		
Family doctor contract				11.757	< 0.001
No	46 (32.9)	3 (8.8)	43 (40.6)		
Yes	94 (67.1)	31 (91.2)	63 (59.4)		
Using smartphone				10.109	< 0.001
No	48 (34.3)	4 (11.8)	44 (41.5)		
Yes	92 (65.7)	30 (88.2)	62 (58.5)		

^a^ Data are *N* (%) unless otherwise stated.

^b^ A threshold of *p* < 0.05 was considered statistically significant.

##### 3.1.1.2 Qualitative Findings of Survey Data

A total of 16 respondents, representing different roles, participated in the interviews.

### 3.2 Attitudes Towards Difficulties in Implementing ‘Health Bank’

The majority of interviewees considered that there existed a relatively high degree of feasibility in addressing the problems with the implementation of ‘Health Bank’, including increasing publicity, lowering digitisation requirements and providing gifts that were more in line with the needs of the population.

My neighbors and friends were willing to join the “Health Bank” after learning about it, but many of them had not heard of it at first until I joined and shared it with them.

Female, 59 years old, participating villager 4

I know there are a lot of suitably priced electronic wearable devices available for different purposes that I think could be used instead of mobile phones.

Male, 41 years old, non‐participating villager 4

I think that by simply exchanging the current gifts such as tissue paper and soap for equivalent health‐related ones such as medical check‐ups, the interest of residents will be greatly enhanced since health is now taken very seriously.

Male, 48 years old, village doctor 2

#### 3.2.1 Routine Data (1164 Participants of ‘Health Bank’)

**Figure 2:**
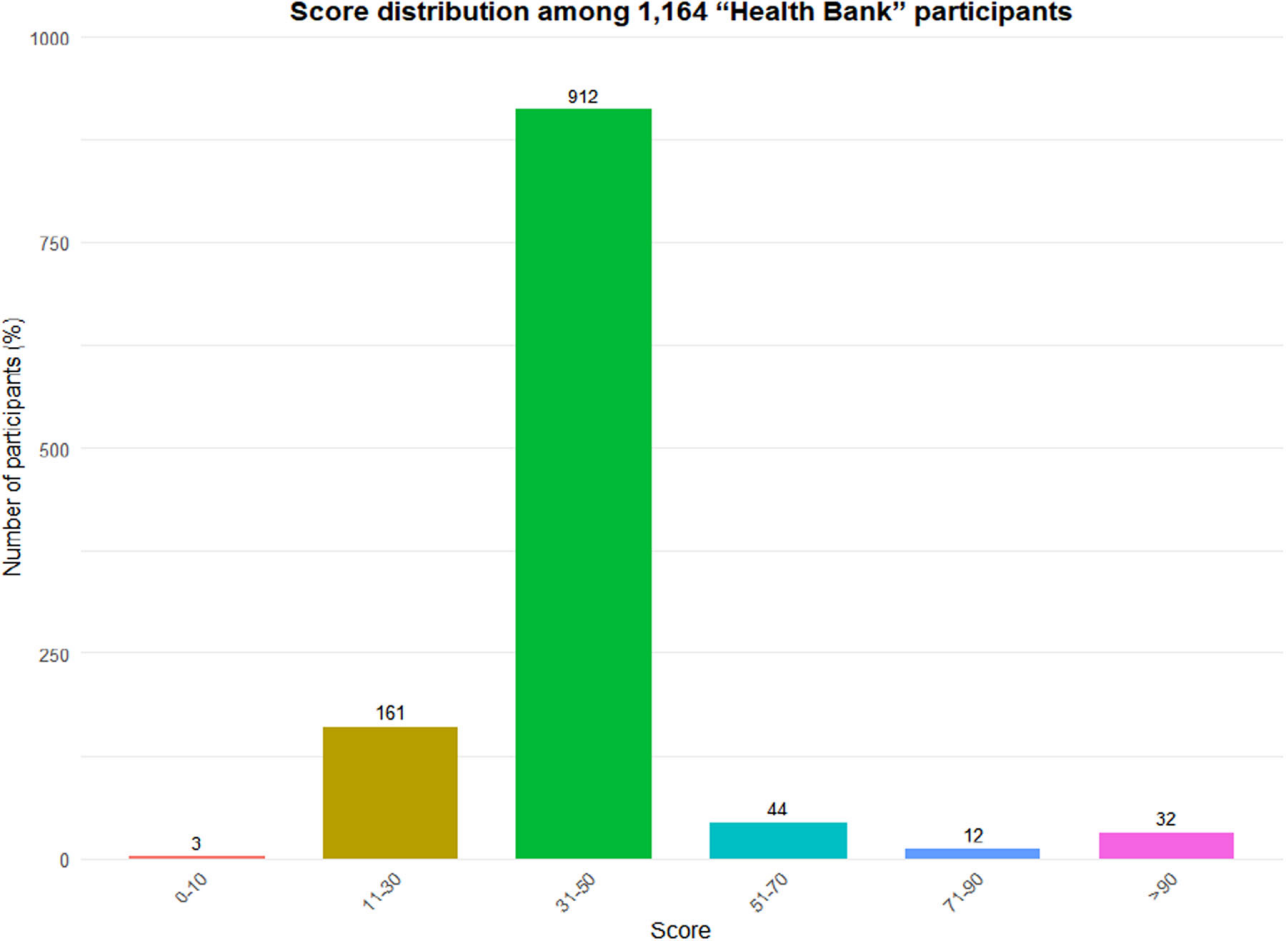
Score distribution among 1164 ‘Health Bank’ participants. Active participants were defined as those with a total score greater than 50.

From January to October 2022, a total of 1164 villagers participated in ‘Health Bank’, representing a coverage rate of 37.1% (1164/3137) across the whole village. As depicted in Figure 2, 88 out of 1164 (7.6%) were active participants, while most of the rest only completed three daily behaviours.

Table 2 provides an overview of the participants in ‘Health Bank’, stratified by different activity levels. Among the active participants, most were women, aged 45–74, and with hypertension or diabetes. Compared with men, women were nearly two times more likely to be active participants (9.8% vs. 5.0%, *p* = 0.002).

**Table 2:** Comparison of demographic characteristics between active and inactive participants of ‘Health Bank’ (*N* = 1164).

Characteristics	Participants in ‘Health Bank’, *N* (%)^a^				
	Total	Active participants^b^	Inactive participants	*χ* ^2^	*p* c
Gender				9.548	0.002***
Male	541 (46.5)	27 (30.7)	514 (47.8)		
Female	623 (53.5)	61 (69.3)	562 (52.2)		
Age group				4.026	0.259
< 45	122 (10.5)	11 (12.5)	111 (10.3)		
45–59	322 (27.7)	30 (34.1)	292 (27.1)		
60–74	498 (42.8)	36 (40.9)	462 (42.9)		
≥ 75	222 (19.1)	11 (12.5)	211 (19.6)		
Hypertension or diabetes				3.624	0.057
Absent	616 (52.9)	38 (43.2)	578 (53.7)		
Present	548 (47.1)	50 (56.8)	498 (46.3)		

^a^ Data are *N* (%) unless otherwise stated.

^b^ Active participants: Active participants were defined as those with a total score greater than 50.

^c^ A threshold of *p* < 0.05 was considered statistically significant.

*** < 0.05.

As shown in Table 3, hypertensive patients were more likely to participate in ‘Health Bank’ (*p* = 0.032), but this was not the case for diabetics and those with both diseases. In addition, participants with hypertension or diabetes did not have a greater probability of monitoring their blood pressure or blood glucose autonomously, respectively.

**Table 3:** Comparison of hypertension and diabetes prevalence between (1) active and inactive participants of ‘Health Bank’ and (2) participants with and without routine blood pressure/glucose monitoring.

Characteristics	Participants in ‘Health Bank’, *N* (%)^a^				
	Total	Active participants^b^	Inactive participants	*χ* ^2^	*p* c
Hypertension				4.593	0.032***
Absent	643 (55.2)	39 (44.3)	604 (47.8)		
Present	521 (44.8)	49 (55.7)	472 (52.2)		
Diabetes				0.561	0.454
Absent	1074 (92.3)	83 (94.3)	991 (92.1)		
Present	90 (7.7)	5 (5.7)	85 (7.9)		
Hypertension or diabetes				5.169	0.140^d^
Normal	616 (52.9)	38 (43.2)	578 (53.7)		
Hypertension only	458 (39.3)	45 (51.1)	413 (38.4)		
Diabetes only	27 (2.3)	1 (1.1)	26 (2.4)		
Hypertension and diabetes	63 (5.4)	4 (4.5)	59 (5.5)		

Characteristics	Participants in ‘Regular blood pressure measurements’, *N* (%)^a^				
	Total	Participants	Non‐participants	*χ* ^2^	*P* c
Hypertension				1.460	0.227
Absent	643 (55.2)	18 (1.1)	625 (55.0)		
Present	521 (44.8)	9 (4.5)	512 (45.0)		

Characteristics	Participants in ‘Regular blood glucose measurements’, *N* (%)^a^				
	Total	Participants	Non‐participants	*χ* ^2^	*P* c
Diabetes				2.316	0.261^d^
Absent	1074 (92.3)	27 (100.0)	1047 (92.1)		
Present	90 (7.7)	0 (0.0)	90 (7.9)		

^a^ Data are *N* (%) unless otherwise stated.

^b^ Active participants: Active participants were defined as those with a total score greater than 50.

^c^ A threshold of *p* < 0.05 was considered statistically significant.

*** < 0.05.

^d^ Fisher's exact test.

### 3.3 Acceptability

#### 3.3.1 Quantitative Findings of Survey Data

**Figure 3:**
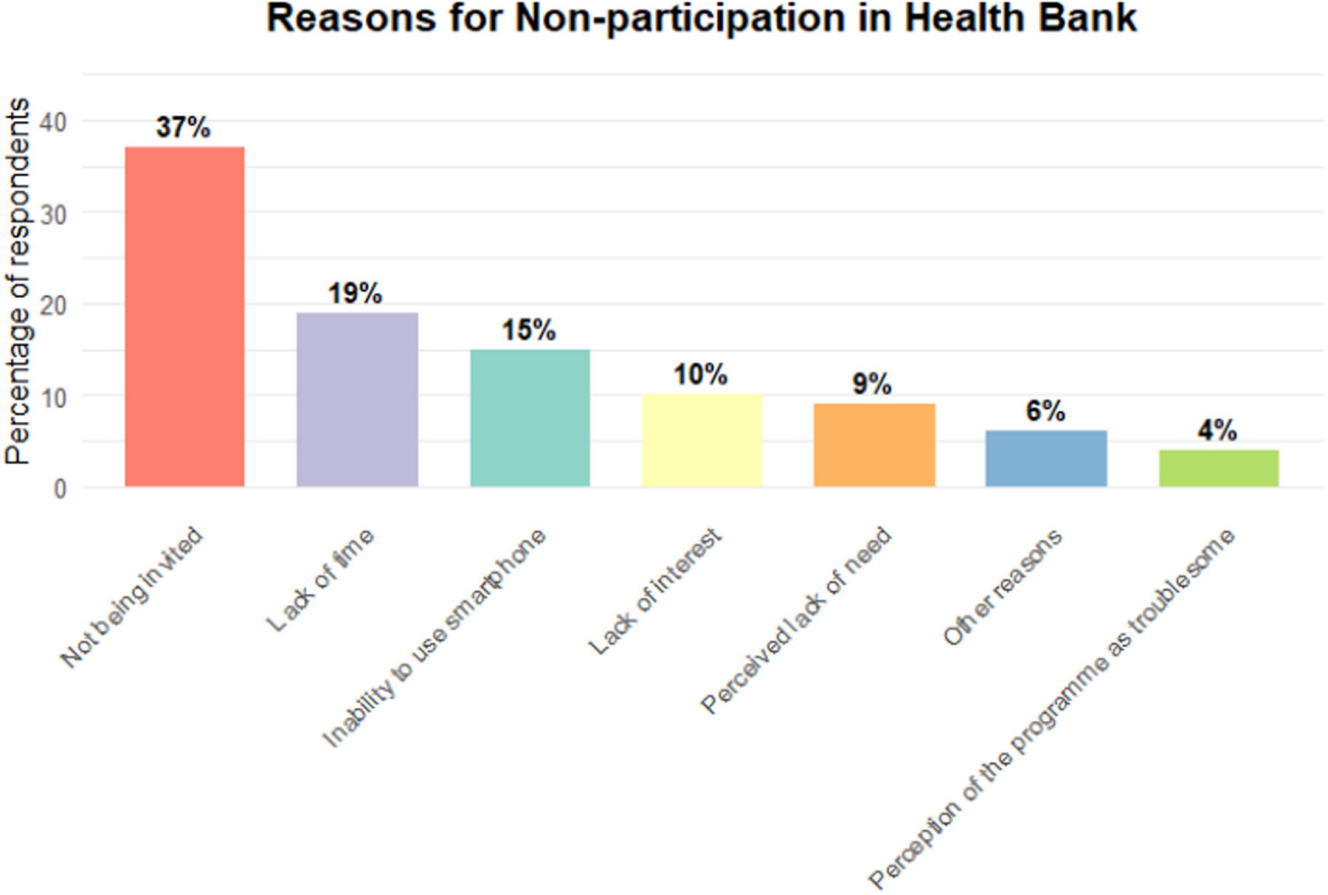
Reasons for non‐participation in ‘Health Bank’.

Of the 140 respondents surveyed, 68 did not participate in ‘Health Bank’ after obtaining related information. Figure 3 illustrates the reasons for non‐participation in ‘Health Bank’, including not being invited (37%), lack of time (19%), inability to use a smartphone (15%), lack of interest (10%), perceived lack of need (9%), perception of the programme as troublesome (4%), and other reasons (6%).

#### 3.3.2 Qualitative Findings of Survey Data

Analysis revealed five major themes related to the acceptability of ‘Health Bank’: (1) broader communication channels; (2) enhanced age‐friendly environments; (3) improvements in evaluation criteria for behaviours included; (4) improvements in gift redemption; and (5) positive repercussions of financial incentives.

#### 3.3.3 Theme 1: Broader Communication Channels

Some villagers who had not participated in the ‘Health Bank’ mentioned that they had never heard of it, but expressed great interest after the investigator introduced the ‘Health Bank’ to them. One village doctor claimed that the activity was not well‐promoted, leading to misunderstandings among some villagers.

I have never heard of the “Health Bank”. But if someone invites me, I would like to participate.

Male, 58 years old, non‐participating villager 1

Is this a government‐sponsored project? I have not been exposed to the relevant news.

Male, 47 years old, non‐participating villager 2

A patient said he did not participate because his wife was worried about being cheated out of money when you mentioned the word “bank”. I thought the government's advocacy was not detailed enough.

Female, 45 years old, village doctor 1

In addition, a village head highlighted the limited communication channels in the village, regarding village events, householder gatherings and various WeChat groups.

The main offline channels only included village events and householder gatherings, while online channels consisted of different kinds of WeChat groups.

Male, 40 years old, village head 1

#### 3.3.4 Theme 2: Enhanced Age‐Friendly Environments

Elderly participants identified the use of mobile phones as a major obstacle to their participation and expressed a desire for appropriate support. A CDC staff also mentioned that villagers always struggled with using electronic devices properly. Some villagers had concerns about the cost of data generated by participating in online activities. Moreover, the requirement to record exercise online in real time caused difficulties for many older participants.

I was willing to participate if others help me operate it on my smartphone.

Female, 54 years old, non‐participating villager 3

It was hard for me to read texts on my smartphone. It would be great if I could participate without it.

Female, 73 years old, non‐participating villager 5

The villagers didn't know how to use the digital instruments such as sphygmomanometer. Usually, they operated in an unregulated manner.

Male, 38 years old, staff of county CDC 1

Participating in online activities consumed a lot of data.

Female, 51 years old, participating villager 2

I had to take my smartphone to participating in square dancing so as to record my exercise automatically into the system, but it was inconvenient.

Female, 47 years old, participating villager 3

#### 3.3.5 Theme 3: Improvements in Evaluation Criteria for Behaviours Included

Participants noted that the criteria for scoring some behaviours were not mandatory. For example, simply keeping the phone screen on a page for 15 seconds without actually reading was considered as having read a science text, which led to a lack of practicality for some behaviours.

I scored a lot by reading health tweets, often by keeping my phone page there while I was cooking.

Female, 51 years old, participating villager 2

#### 3.3.6 Theme 4: Improvements in Gift Redemption

**Figure 4:**
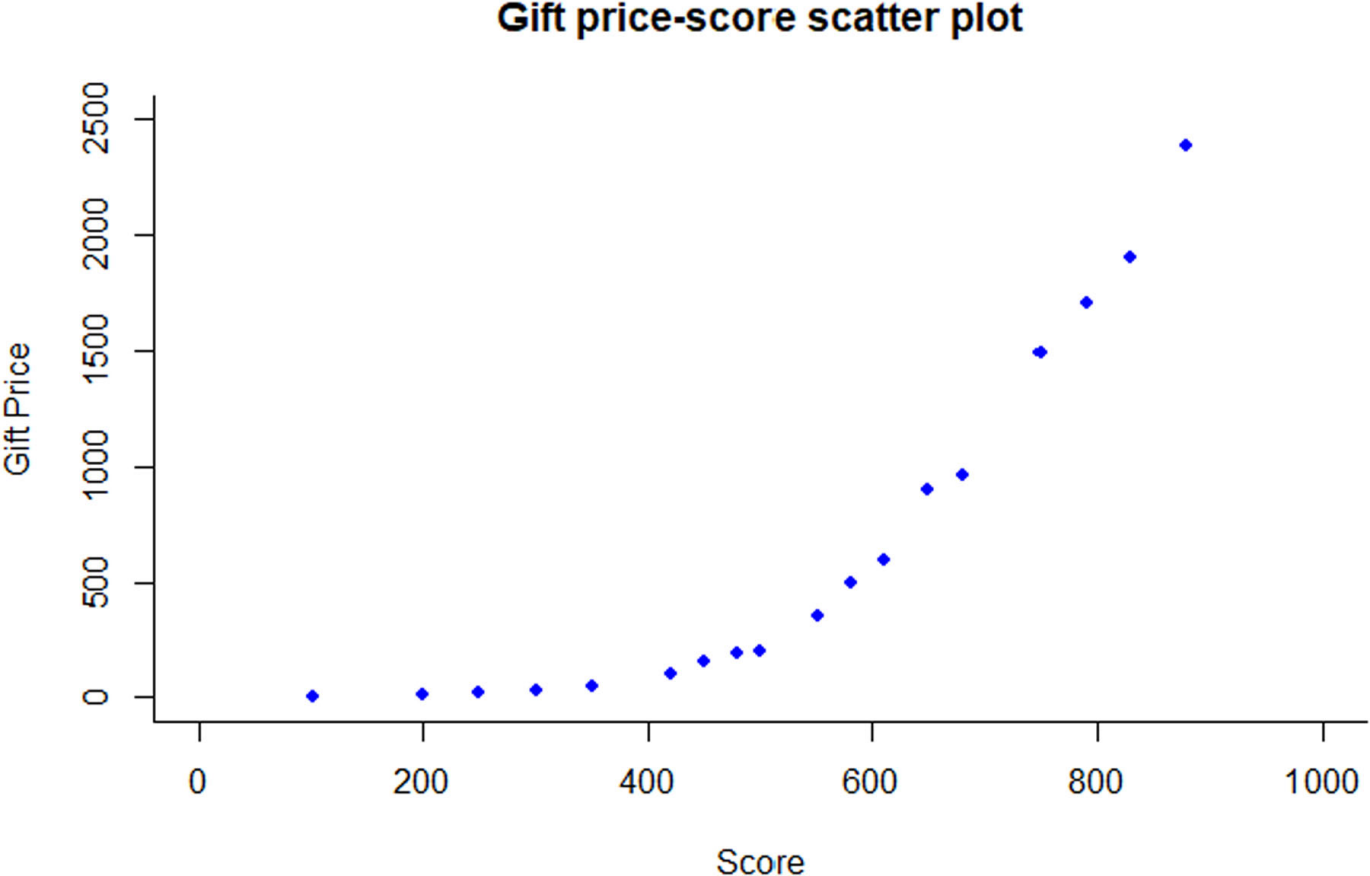
Gift price‐score scatter plot.

Participants reported that the scores required to redeem higher‐value gifts were hard to reach, and the accessibility of gifts needed to be improved. As shown in the gift price‐score scatter plot (Figure 4), the rising trend of gift value and corresponding required scores cannot be accurately matched, indicating that most participants were only able to redeem inexpensive gifts. In addition, participants expressed a desire to include more gifts of non‐physical health services, such as free physical exams.

The score for the gift I wanted was too high. I only had approximately 100 points so that I gave up.

Male, 72 years old, participating villager 1

I had chronic diseases and I needed to have an annual physical exam every year. Could I get regular free physical exams as a gift if I participate?

Female, 59 years old, participating villager 4

The villagers would like to add specialist consultations from higher level hospitals to the gift pool.

Male, 50 years old, village head 2

#### 3.3.7 Theme 5: Positive Repercussion of Financial Incentives

Some villagers expressed great satisfaction with the ability to obtain gifts while participating in the activities. The village head also mentioned that gifts were considerably helpful to motivate villagers to participate in ‘Health Bank’.

We think this platform is great, not only can we be encouraged to exercise, but we can also get gifts.

Female, 48 years old, participating villager 5

There were many villagers who came mainly for the gifts. Villagers were much more motivated to participate because of the gifts.

Male, 40 years old, village head 1

## 4 Discussion

Our findings suggested that the financial incentive intervention was both well‐accepted and relatively feasible. Furthermore, tailored modifications are crucial for subsequent large‐scale studies, including implementing more reasonable financial incentives through incorporating behavioural economic theories and scaling up the digital platform application.

In terms of feasibility, nearly half of the villagers were involved in ‘Health Bank’, and this finding is consistent across survey data and routine data. However, the proportion of active participants was relatively low, and several challenges remained with the intervention. First, a notable issue was the gender disparity, with more women actively participating in activities, which is consistent with previous findings indicating that women are more likely to engage in social activities [40]. Future trials should consider this issue by adjusting the design to attract more men to balance the participation rate by gender. One potential approach involves shifting from an individual‐based model to a family‐based model, in which points earned by all household members are accumulated into a shared family account. This structure functions as a form of ‘group contingency’ (GC). GC interventions are characterised by the application of one or more contingency components, such as criteria, behaviours or reinforcement, to the performance of one or more members of a group. These processes combine implementer‐delivered and peer‐mediated reinforcement to promote behavioural change across all group members and often encourage cooperation among participants [41]. Additional social consequences may be presented by group members to ensure the reinforcement criterion is met [42]. Besides, ‘Shared goals’ is thought to foster connections between individuals and thus accelerate the goal attainment [43]. In addition, the coverage of the digital platform can be further expanded by introducing a referral mechanism, where active female participants earn scores by referring other inactive male family members or other villagers to participate in ‘Health Bank’. Second, the digital divide has significantly reduced the participation of elderly people, especially in rural China, where the proportion of the elderly is extremely high [44]. To improve the feasibility for this population, identity card verification and wearable devices connected to digital platforms could be applied. Village doctors and volunteers could also provide assistance to enable automatic acquisition and an offline record of behavioural information. Third, a majority of participants gained 50 scores through three behaviours, including receiving a Covid‐19 vaccination, contracting a family doctor, and undergoing an annual physical exam, and then no longer participated in other behaviours. More tailored measures are needed to facilitate or attract these participants to be involved in other expected behaviours.

In terms of acceptability, the results were generally favourable. Among the 68 survey respondents who chose not to participate in the ‘Health Bank’, only 10% cited a lack of interest, while 9% mentioned not having a need for it. The major reasons given for non‐participation were objective factors, such as time constraints, concerns about personal information security, and data consumption. To assuage the villagers' concerns, it is necessary for village heads, village doctors and opinion leaders among the participants to actively share their experience of utilising the digital platform and assure villagers of the absolute security of user information. Additionally, to reduce the potentially high data consumption, the government could collaborate with Internet service providers and telecommunications companies to introduce free data available only to the digital platform for participants' use.

As for the future directions of financial incentives, a significant issue identified through the qualitative interviews was the corresponding setting of scores and gifts. In our study, we found that most residents only redeemed low‐value gifts rather than choosing to accumulate scores over time to redeem higher‐value ones. Participants might feel the required score for the high‐value gift was too high to get and consider they were unable to achieve it and, therefore, easily give up. One potential approach is the application of probability‐based cost management (PB‐CM), also known as the fishbowl method, as proposed by Nancy Petry et al. This method aims to reduce costs by incorporating probability and variability [45]. Future implementations could consider assigning different probabilities of winning higher‐value prizes to participants based on their accumulated points. For instance, even participants with lower point totals would retain a small chance of winning a more valuable reward. Such a design may enhance participant motivation and sustained engagement. Empirically, financial incentives designed using concepts from behavioural economics have been shown to be effective for promoting health behaviour change [46]. Interventions designed according to behavioural economic incentives could capitalise on predictable cognitive tendencies, such as present bias, loss aversion, regret aversion, the propensity to overestimate low‐probability events, and heightened responsiveness to variable reinforcement schedules compared with fixed rewards. These approaches encompass a broader array of influences on human decision‐making than monetary value alone [46]. Several intervention trials have demonstrated that economic incentives designed using behavioural economics principles can achieve favourable outcomes in promoting health behaviours such as smoking cessation, alcohol reduction, vaccination uptake and obesity prevention [47, 48, 49, 50]. Furthermore, qualitative results suggested that a participatory approach can be applied, which implies that everyone who has a stake in the intervention has a voice and that all participation should be welcomed and respected [51]. For example, gifts that fit the specific needs of participants are important.

## 5 Limitations

There are several limitations in this study. First, as an evaluation of a single‐group pilot intervention, the study primarily focused on assessing the feasibility and acceptability of the intervention rather than evaluating its actual impact on healthy behaviours. Thus, its efficacy can be obtained through future randomised controlled trials. Second, this pilot intervention was conducted at a single site with a relatively small sample size, which may limit the generalisability of the findings to a broader population. Third, the participants were self‐selected volunteers, which introduces the potential for self‐selection bias. Individuals motivated to join a health intervention pilot may exhibit higher baseline engagement and health literacy, potentially leading to an overestimation of feasibility and acceptability metrics. Villagers with comprehension difficulties (e.g., illiteracy) were excluded, and these individuals should be prioritised as intervention targets. Future intervention tools are needed to bridge this gap. However, as this was a pilot study aimed primarily at assessing feasibility and refining the intervention protocol, recruiting a willing cohort was pragmatically necessary. Fourth, we employed purposive sampling in the qualitative component of this study, which may limit the generalisability of the qualitative findings. Nevertheless, this approach allowed for targeted recruitment aligned with the research objectives and facilitated the collection of contextually rich data during the preliminary exploratory phase. Furthermore, purposive sampling enhanced the operational feasibility of the study and provided nuanced insights pertinent to the initial research questions [52].

## 6 Conclusion

The results demonstrate that implementing an incentive‐based implementation strategy through a digital platform to promote health behaviours in a rural setting is feasible and acceptable. Our findings emphasise the need for larger randomised controlled trials to evaluate the effectiveness of the intervention. In addition, behavioural theories and behaviour change technique‐taxonomy (BCTT) are recommended to build a more reasonable mechanism of financial incentive interventions and to identify the active ingredients and their efficacy among the components of interventions [53]. Meanwhile, it is also significant to monitor whether incentives may unintentionally cause negative consequences [54]. This study provides a promising foundation for further research and development of incentive‐based interventions to enhance healthy behaviours in rural communities.

## Author Contributions

**Bo Yan:** data analysis, manuscript drafting. **Zhenke He:** data analysis, manuscript drafting. **Ran Zhang:** critical review of article, article revision. **Bin Shao:** data collection. **Shuai Zheng:** data collection. **Xiaomin Wang:** study conception, study design, critical review of article, article revision. **Xudong Zhou:** study conception, study design, critical review of article, article revision. All authors reviewed and edited the manuscript and approved the final version of the manuscript.

## Ethics Statement

This is an evaluation study of ‘Health Bank’ which was conducted in collaboration with the local government. ‘Health Bank’ is a digital platform developed and operated by the local CDC, which has obtained written informed consent from all participants. In China, data provided by health sectors can be made accessible for research purposes, and these data were allowed to be used to identify, invite and contact potential participants relevant to the study. Therefore, this study was granted exemption from formal ethical approval on the basis of the evaluation not in any way limiting the autonomy of the participants regarding their decision to participate in ‘Health Bank’. The requirement for ethical approval was waived by the Ethics Committee of the School of Public Health at Zhejiang University because of the retrospective nature of the study. Ethical principles were adhered to throughout the study, ensuring the confidentiality and privacy of participant information. The study was conducted in accordance with the related guidelines and regulations for a non‐clinical trial.

## Consent

The local CDC has obtained written informed consent from all participants.

## Conflicts of Interest

The authors declare no conflicts of interest.

## Supporting information

Supplemental materials for this article are available online.

## Data Availability

The datasets are available from the corresponding author upon reasonable request.
