# Supplementary material for: Feasibility and Acceptability of an Incentive‐Based Intervention for Health Behaviour Change Among Rural Residents in China: A Mixed‐Methods Evaluation
Source: Health Expect. 2025 Dec 11;28(6):e70518. doi: 10.1111/hex.70518 (PMC12698508; doi:10.1111/hex.70518)
Supplement: Supplementary file 1 — Supplemental materials for this article are available online. [file HEX-28-e70518-s001.docx]

# **Supplementary Material 1**

Table S1. Scores Setting and Recording Rules of “Health Bank”

| Activity | Route | Score | Interpretation |
| --- | --- | --- | --- |
| Regular blood pressure measurements | Off-line | 10 | Measured at the Health Bank service station, 10 scores/quarter |
| Regular blood glucose measurements | Off-line | 10 | Measured at the Health Bank service station, 10 scores/quarter |
| Regular BMI measurements | Off-line | 15 | Measured at the Health Bank service station, 15 scores/quarter |
| Annual physical exam | Off-line | 20 | Organized by medical institution, 20 scores/time |
| COVID-19 vaccine uptake | Off-line | 20 | Organized by medical institution, 20 scores/time |
| Regular attendance at health lectures | Off-line | 20 | Organized by government and medical institution, 20 scores/time |
| Family doctor contract | Off-line | 30 | Organized by government and medical institution, 20 scores/time |
| Daily exercise | On-line | 5 | Exercising more than 6,000 steps on 18 or more days per month, 5 scores/month |
| Routine testing of health-related knowledge | On-line | 5 | Taking the quiz by smartphone, 5 scores/period |
| Reading of tweets with health-related topics | On-line | 1 | Reading the information by smartphone, 1 score/item |

Table S2. Gift Redemption Rules of “Health Bank”

| Type | Gift | Score | Price(RMB) |
| --- | --- | --- | --- |
| Electronic product | Electric bicycle | 880 | 2388 |
|  | Sports watch | 750 | 1488 |
|  | Treadmill | 680 | 968 |
|  | Foldable bicycle | 650 | 900 |
|  | Electric massager | 610 | 598 |
|  | Smartphone | 580 | 500 |
|  | Sports bracelet | 550 | 350 |
|  | Sphygmomanometer | 480 | 188 |
|  | Electronic scale | 420 | 98 |
| Health service | Physical examination package | 650 | 918 |
|  | Coupon for CT and DR | 500 | 200 |
| Household goods | Television | 830 | 1899 |
|  | Electric refrigerator | 790 | 1700 |
|  | Electric cooker | 550 | 350 |
|  | Juicer | 450 | 158 |
|  | Umbrella | 350 | 50 |
|  | Thermos cup | 350 | 50 |
|  | Towel | 300 | 28 |
|  | Tissue | 250 | 18 |
|  | Toothpaste | 200 | 12 |
|  | Detergent | 100 | 6 |

# **Supplementary Material 2**

**Questionnaire on participation status of “Health Bank”(for participants)**

**Part 1 Basic information**

Q1. Gender?

① Male ② Female

Q2. Age ______

Q3. Household size?

① 1 ② 2 ③ 3 ④ 4 ⑤ 5 ⑥ other ______

Q4. Average monthly household income (RMB)？

① ＜2000 ② 2001-4000 ③ 4001-6000

④ 6001-8000 ⑤ 8001-10000 ⑥ >10000

Q5. Educational level?

① Never attended school ② Primary school ③ Junior high school

④ High school/Secondary special school ⑤ Undergraduate/Junior college

⑥ Postgraduate and above

Q6. Have you been involved in family doctor contract services？

① Yes ② No

Q7. How long does it take you to walk from your home to the Community Service Centre?

① 5 minutes or less ② 5-10 minutes ③ 10-15 minutes

④ 15-20 minutes ⑤ 20-25 minutes ⑥ 25 minutes or above

Q8. Are you hypertensive and/or diabetic?

① Yes ② No (Skip to Q9) ③ Don't know (Skip to Q9)

Q8.1. If yes, have you participated in regular blood pressure or glucose monitoring?

① Yes (Skip to Q9) ② No

Q8.2. If not participated, the reason is: __________________________________

Q9. Do you usually use a smartphone?

① Yes (Skip to Q10) ② No

Q9.1. If you don't use a smartphone, the reason is: __________________________________

① Don't have a smartphone ② Have a smartphone but cannot use it ③ Other ______

Q10. I am very concerned about my health.

① Strongly disagree ② Disagree ③ Unsure ④ Comparatively agree ⑤ Strongly agree

Q11. I can manage my own health.

① Strongly disagree ② Disagree ③ Unsure ④ Comparatively agree ⑤ Strongly agree

Q12. I'm worried I'm going to get a chronic disease.

① Strongly disagree ② Disagree ③ Unsure ④ Comparatively agree ⑤ Strongly agree

Q13. I consider my health better than others.

① Strongly disagree ② Disagree ③ Unsure ④ Comparatively agree ⑤ Strongly agree

Q14. I think I'm at a higher risk of getting chronic diseases than others.

① Strongly disagree ② Disagree ③ Unsure ④ Comparatively agree ⑤ Strongly agree

Q15. Do you participate in “Health Bank”?

① Yes ② No

**Part 2 Participation status of “Health Bank”**

Q16. If participating, points awarded so far in 2022: ______.

Q17. How did you hear about the “Health Bank”?

① Village doctors ② Village cadres ③ Relatives, neighbors, or friends

④ CDC staff ⑤ Billboards ⑥ Other ______

Q18. What is the main purpose of your participation in “Health Bank”?

① Earn points for prizes ② Enhance health literacy ③ Access convenient medical services

④ Other ______

Q19. How often will you participate in “Health Bank” in 2022?

① Never ② Seldom ③ Sometimes ④ Often ⑤ Always

Q20. Which of the activities offered in “Health Bank” are you satisfied with? (Multiple choice possible)

① Regular blood pressure/glucose/BMI measurements ② health lectures

③ health-related topics ④ Daily exercise ⑤ Annual physical exam

⑥ Routine testing of health-related knowledge ⑦ Other __________ ⑧ None

Q21. Which of the activities offered in “Health Bank” are you not satisfied with? (Multiple choice possible)

① Regular blood pressure/glucose/BMI measurements ② health lectures

③ health-related topics ④ Daily exercise ⑤ Annual physical exam

⑥ Routine testing of health-related knowledge ⑦ Other __________ ⑧ None

Q22. What activities do you think could be added to the “Health Bank” that you feel is needed？

_______________________________________________________________________________

Q23. What are the factors that normally prevent you from participating in activities? (Multiple choice possible)

① Lack of time ② Lack of interest ③ Lack of incentives due to unreasonable point rules ④ Difficulty in operation ⑤ No relevant needs ⑥ No use of smartphones ⑦ Other ______

Q24. Did you already have a certain number of points, but don't insist on participating?

① Yes ② No (Skip to Q25)

Q24.1. If so, why?

① Lack of time ② Lack of interest ③ Lack of incentives due to unreasonable point rules ④ Difficulty in operation ⑤ No relevant needs ⑥ No use of smartphones ⑦ Other ______

Q25. What do you think needs to be done to improve for the above reasons that prevent you from participating?

_______________________________________________________________________________

Q26. Have you ever shared “Health Bank” with others or invited others to participate in “Health Bank”?

① Yes (Skip to Q27) ② No

Q26.1. If not, why?

_______________________________________________________________________________

**Part 3 Suggestions & comments on “Health Bank”**

Q27. If other family members are enrolled in “Health Bank” with your help, you will be given a certain amount of points each year as a reward. Would you be willing to do so?

① Yes (Skip to Q28) ② No ③ Don't know (Skip to Q28)

Q27.1. If not, why?？

_______________________________________________________________________________

Q28. If you help elderly people who live alone to participate in “Health Bank” or assist them to participate in activities, you will be given a certain amount of points each year as a reward. Would you be willing to do so?

① Yes (Skip to Q29) ② No ③ Don't know (Skip to Q29)

Q28.1. If not, why?

_______________________________________________________________________________

Q29. If you are in charge of the operation of the “Health Bank” and the design of activities, you will be given a certain amount of points each year as a reward. Would you be willing to do so?

① Yes (Skip to Q30) ② No ③ Don't know (Skip to Q30)

Q29.1. If not, why?

_______________________________________________________________________________

Q30. What are your suggestions for increasing participation in “Health Bank”?

_______________________________________________________________________________

# **Supplementary Material 3**

**Questionnaire on participation status of “Health Bank”(for non-participants)**

**Part 1 Basic information**

Q1. Gender?

① Male ② Female

Q2. Age ______

Q3. Household size?

① 1 ② 2 ③ 3 ④ 4 ⑤ 5 ⑥ other ______

Q4. Average monthly household income (RMB)？

① ＜2000 ② 2001-4000 ③ 4001-6000

④ 6001-8000 ⑤ 8001-10000 ⑥ >10000

Q5. Educational level?

① Never attended school ② Primary school ③ Junior high school

④ High school/Secondary special school ⑤ Undergraduate/Junior college

⑥ Postgraduate and above

Q6. Have you been involved in family doctor contract services？

① Yes ② No

Q7. How long does it take you to walk from your home to the Community Service Centre?

① 5 minutes or less ② 5-10 minutes ③ 10-15 minutes

④ 15-20 minutes ⑤ 20-25 minutes ⑥ 25 minutes or above

Q8. Are you hypertensive and/or diabetic?

① Yes ② No (Skip to Q9) ③ Don't know (Skip to Q9)

Q8.1. If yes, have you participated in regular blood pressure or glucose monitoring?

① Yes (Skip to Q9) ② No

Q8.2. If not participated, the reason is: __________________________________

Q9. Do you usually use a smartphone?

① Yes (Skip to Q10) ② No

Q9.1. If you don't use a smartphone, the reason is: __________________________________

① Don't have a smartphone ② Have a smartphone but cannot use it ③ Other ______

Q10. I am very concerned about my health.

① Strongly disagree ② Disagree ③ Unsure ④ Comparatively agree ⑤ Strongly agree

Q11. I can manage my own health.

① Strongly disagree ② Disagree ③ Unsure ④ Comparatively agree ⑤ Strongly agree

Q12. I'm worried I'm going to get a chronic disease.

① Strongly disagree ② Disagree ③ Unsure ④ Comparatively agree ⑤ Strongly agree

Q13. I consider my health better than others.

① Strongly disagree ② Disagree ③ Unsure ④ Comparatively agree ⑤ Strongly agree

Q14. I think I'm at a higher risk of getting chronic diseases than others.

① Strongly disagree ② Disagree ③ Unsure ④ Comparatively agree ⑤ Strongly agree

Q15. Do you participate in “Health Bank”?

① Yes ② No

**Part 2 Suggestions & comments on “Health Bank”**

Q16. Have you heard of “Health Bank”?

① Yes ② No (Skip to Q25)

Q17. If yes, how did you hear about the “Health Bank”?

① Village doctors ② Village cadres ③ Relatives, neighbors, or friends

④ CDC staff ⑤ Billboards ⑥ Other ______

Q18. Which of the activities offered in “Health Bank” are you satisfied with? (Multiple choice possible)

① Regular blood pressure/glucose/BMI measurements ② health lectures

③ health-related topics ④ Daily exercise ⑤ Annual physical exam

⑥ Routine testing of health-related knowledge ⑦ Other __________ ⑧ None

Q19. Which of the activities offered in “Health Bank” are you not satisfied with? (Multiple choice possible)

① Regular blood pressure/glucose/BMI measurements ② health lectures

③ health-related topics ④ Daily exercise ⑤ Annual physical exam

⑥ Routine testing of health-related knowledge ⑦ Other __________ ⑧ None

Q20. What activities do you think could be added to the “Health Bank” that you feel is needed? _______________________________________________________________________________

Q21. Does your family participate in “Health Bank”?

① Yes ② No

Q22. Have you ever been invited to participate in “Health Bank”?

① Yes ② No (Skip to Q23)

Q22.1. If so, why did you refuse to participate? (Multiple choice possible)

① Lack of time ② Lack of interest ③ Lack of incentives due to unreasonable point rules ④ Difficulty in operation ⑤ No relevant needs ⑥ No use of smartphones ⑦ Other ______

Q22.2. What do you think needs to be done to improve for the above reasons that prevent you from participating?

_______________________________________________________________________________

Q23. If you were invited by a family member, neighbour or friend to participate in a health bank project, would you be willing to do so?

① Yes (Skip to Q24) ② No

Q23.1. If still not willing to participate, why?

_______________________________________________________________________________

Q24. What are your suggestions for increasing participation in “Health Bank”?

_______________________________________________________________________________

Q25. If there is a programme where you can earn points through a series of healthy behaviors and then exchange them for various gifts of varying values, would you be willing to participate?

① Yes ② No

Q25.1. If still not willing to participate, why?

_______________________________________________________________________________

# **Supplementary Material 4**

**Outline of qualitative interview**

**Theme 1: Broader communication channels**

Q1. Have you heard of “Health Bank”?

Q2. What channels do you usually use to get information from the government?

Q3. What are the main channels through which the government and the health sector promote the “health bank”? (For government staff)

**Theme 2: Enhanced age-friendly environments**

Q1. Do you have any operational or technical difficulties in using the "Health Bank" platform?

Q2. When you have trouble using digital devices such as smartphones and blood pressure monitors, where do you turn for help? Do these difficulties discourage you from continuing to participate?

Q3. Which of the activities offered in “Health Bank” are you satisfied with or not?

Q4. What do you think needs to be done to improve for the reasons that prevent you from participating?

**Theme 3: Improvements in evaluation criteria for behaviors included**

Q1. Do you spend time reading the health education materials provided in the “Health Bank” APP every day? Usually at what time of day?

Q2. What will the government do about the fact that some activities’ scores can be earned too easily and fail to achieve their intended results? (For government staff)

**Theme 4: Improvements in gift redemption**

Q1. Do you think the setting of points and the corresponding gifts are reasonable?

Q2. Do you find it difficult to get the gift you want?

Q3. What other gifts do you think you can add that you think are in demand?

Q4. How will the government improve the gift pool in the future? (For government staff)

**Theme 5: Positive repercussion of financial incentives**

Q1. Do financial incentives appeal to you?

Q2. Does the introduction of economic incentives increase participation in healthy behaviors?

Q3. What activities do you think could be added to the “Health Bank” that you feel is needed?

Q4. What are your suggestions for increasing participation in “Health Bank”?
